# Reactivation and Lytic Replication of Kaposi’s Sarcoma-Associated Herpesvirus: An Update

**DOI:** 10.3389/fmicb.2017.00613

**Published:** 2017-04-20

**Authors:** Kawalpreet K. Aneja, Yan Yuan

**Affiliations:** Department of Microbiology, University of Pennsylvania School of Dental Medicine, PhiladelphiaPA, USA

**Keywords:** Kaposi’s sarcoma-associated herpesvirus (KSHV), human herpesvirus 8 (HHV-8), lytic replication, viral reactivation, Rta

## Abstract

The life cycle of Kaposi’s sarcoma-associated herpesvirus (KSHV) consists of two phases, latent and lytic. The virus establishes latency as a strategy for avoiding host immune surveillance and fusing symbiotically with the host for lifetime persistent infection. However, latency can be disrupted and KSHV is reactivated for entry into the lytic replication. Viral lytic replication is crucial for efficient dissemination from its long-term reservoir to the sites of disease and for the spread of the virus to new hosts. The balance of these two phases in the KSHV life cycle is important for both the virus and the host and control of the switch between these two phases is extremely complex. Various environmental factors such as oxidative stress, hypoxia, and certain chemicals have been shown to switch KSHV from latency to lytic reactivation. Immunosuppression, unbalanced inflammatory cytokines, and other viral co-infections also lead to the reactivation of KSHV. This review article summarizes the current understanding of the initiation and regulation of KSHV reactivation and the mechanisms underlying the process of viral lytic replication. In particular, the central role of an immediate-early gene product RTA in KSHV reactivation has been extensively investigated. These studies revealed multiple layers of regulation in activation of RTA as well as the multifunctional roles of RTA in the lytic replication cascade. Epigenetic regulation is known as a critical layer of control for the switch of KSHV between latency and lytic replication. The viral non-coding RNA, PAN, was demonstrated to play a central role in the epigenetic regulation by serving as a guide RNA that brought chromatin remodeling enzymes to the promoters of RTA and other lytic genes. In addition, a novel dimension of regulation by microPeptides emerged and has been shown to regulate RTA expression at the protein level. Overall, extensive investigation of KSHV reactivation and lytic replication has revealed a sophisticated regulation network that controls the important events in KSHV life cycle.

## Introduction

Herpesviruses are extremely successful parasites. Once infected, the individual carries the virus for the rest of life. The success is attributable to the abilities of herpesvirus to (i) efficiently enter host cells, (ii) decisively establish life-long latent infection, and (iii) effectively reactivate upon stimulation from which progeny viruses can be propagated for dissemination within the host and transmission between individuals. Pharmaceutical intervention of any of the three steps can break the chain of viral life cycle and serve as antiviral strategies for treatment of virally associated human diseases or cancers. Achieving this goal relies on a thorough comprehension of the processes in the viral life cycle and the underlying mechanisms.

Kaposi’s sarcoma-associated herpesvirus (KSHV), also termed human herpesvirus type 8 is a member of the human γ-herpesvirus family. It is an etiological agent of Kaposi’s sarcoma (KS), a common AIDS-associated malignancy (Chang et al., 1994), as well as two lymphoproliferative diseases, namely primary effusion lymphoma and multicentric Castleman’s disease (Antman and Chang, 2000; Giffin and Damania, 2014). Unique among herpesviruses, the lytic cycle of KSHV is not only required for the production of progeny viruses, but also contribute to the viral oncogenesis including the development of KS. Studies of KSHV in the last two decades have led to a great comprehension about reactivation and lytic replication of the virus including some common mechanisms shared by all herpesviruses and the unique features that contribute to the special life cycle of KSHV or gamma-herpesviruses as well as diseases specifically associated with KSHV. This review updates the current knowledge of the reactivation of latently infected KSHV and its lytic replication.

## Two Phases of KSHV Life Cycle and their Biological Significance

As a herpesvirus, the life cycle of Kaposi’s sarcoma-associated herpesvirus (KSHV) consists of two phases, latent and lytic (Renne et al., 1996; Miller et al., 1997). In immunocompetent individuals, KSHV establishes latent infection following an acute infection. Latent infection is characterized by expression of only a few of viral genes (termed latent genes) and no production of infectious virions. Multiple copies of viral genome are maintained as extrachromosomal episomes and are replicated in synchrony with cell division (Ballestas et al., 1999). When latency is disrupted, KSHV switches to a lytic life cycle, where the virus expresses most or all of its genes, viral DNA is amplified by the rolling cycle mechanism that generates long head-to-tail concatemers of viral genomes, and progeny virions are assembled and released from the cells (Renne et al., 1996; Gradoville et al., 2000; Lin et al., 2003).

What is the biological significance for a virus to have both latency and lytic life cycles? KSHV has coevolved with its mammalian hosts for millions of years (McGeoch and Davison, 1999) and development of lifetime latency in host is an outcome of the compromises from both sides. It is not in the evolutionary interest of viruses to kill or seriously hurt their residence because viruses need hosts to be healthy in order to support their survival and reproduction. On the other hand, host immune surveillance is competent enough to keep viruses from being harmful (Bhatt and Damania, 2013). T cell responses to KSHV have been studied mostly in Kaposi’s sarcoma (KS) patients and asymptomatic KSHV carriers and such responses to several lytic and latent viral proteins have been detected and demonstrated to be functionally cytotoxic *in vitro*. Both CD4 and CD8 T cell responses have been detected. For example, CD8 T cell responses were found against a broad spectrum of immediate-early (IE), delayed-early (DE), and late gene products like ORF8, ORF22, ORF25, ORF26, and ORF57 (Wang et al., 2001; Lambert et al., 2006; Lepone et al., 2010) and CD4 responses to ORF57, ORF73, K12, K15 and K8.1 (Guihot et al., 2006; Robey et al., 2010). The most compelling evidence for the role of T cell immunity in controlling KSHV reactivation came from Myoung and Ganem (2011) who showed that when mixed culture of human tonsillar B cells and activated T cells were exposed to KSHV, latent infection could be established in B cells with little spontaneous virus production. However, depletion of T cells from the mix or treating the mixed culture with immune suppressants greatly enhanced spontaneous lytic production, demonstrating the importance of T cell immunity in containing KSHV reactivation.

In parallel to T cell suppression of KSHV reactivation, KSHV pathogenicity, such as KS development, is also under the control of T cell immunity. Low CD4 T cell counts in HIV-infected individuals are directly associated with the incidence of KS, which can spontaneously regress with immune reconstitution through highly active antiretroviral therapy (HAART) (Lebbe et al., 1998; Jones et al., 2000; Cattelan et al., 2001; Pellet et al., 2001; Wilkinson et al., 2002; Casper et al., 2004a; Mocroft et al., 2004).

Owing to the effective immune surveillance, especially T cell immunity, to KSHV, establishment of latency in an infected individual is a wise strategy for the viruses to escape immune surveillance and maintain persistent infection. However, reactivation from latency and switch to lytic viral replication cycle is necessary for the viruses to propagate in the individual and spread to other individuals.

## Initiation of KSHV Reactivation and Switch from Latent to Lytic Replication

The balance between KSHV latent and lytic viral life cycle is under the control of sophisticated mechanisms that make it possible for a herpesvirus to get in and out of its host efficiently and freely. In addition to serving for viral propagation, the KSHV lytic life cycle also plays important roles for viral pathogenicity. In KS lesions, most spindle cells are latently infected with KSHV, but a small percentage of these cells undergo spontaneous lytic replication (Zhong et al., 1996; Staskus et al., 1997; Sun et al., 1999). Viral propagation and release of nascent KSHV particles is crucial to sustaining the population of latently infected cells that otherwise would be quickly lost by segregation of latent viral episomes as spindle cells divide (Grundhoff and Ganem, 2004). Thus, KSHV lytic replication and constant infection to fresh cells are crucial for viral tumorigenesis.

### Cellular Factors and Signaling

**FIGURE 1:**
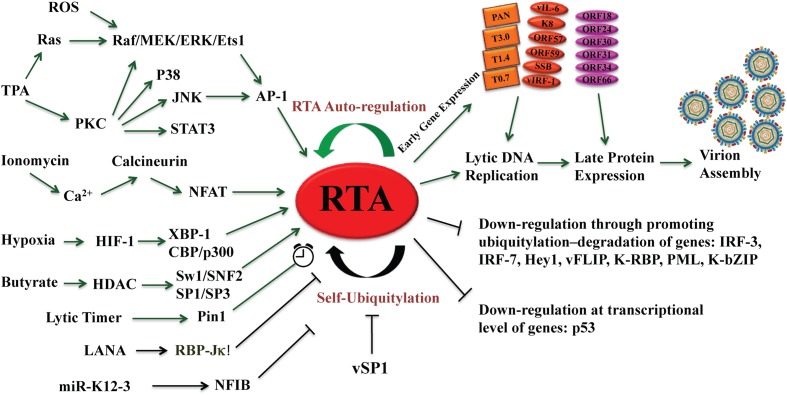
**The central role of RTA in reactivation of Kaposi’s sarcoma-associated herpesvirus (KSHV) from latency leading to lytic cycle.** Different types of signaling pathways and several physiological factors including hypoxia, oxidative stress, and reactive oxygen species (ROS) can disrupt KSHV latency and reactivate the virus by activating the RTA promoter. RTA activation sets up the cascade of gene expression leading to viral lytic replication and virion assembly. RTA auto-regulates its expression at the transcriptional level by using cellular RBP-Jκ notch signaling pathway repressor and by Oct-1, as well as at post-translational level by self-ubiquitylation. The microPeptide vSP-1 blocks the self-ubiquitylation of RTA and promotes viral lytic replication. Green arrows represent activation and black arrows show inhibition.

The reactivation of KSHV can be initiated artificially by treating latently infected cells with certain chemicals such as 12-O-Tetradecanoyl-phorbol-13-acetate (TPA) and sodium butyrate (Miller et al., 1997). These chemicals have been used as very handy tools in the research on the biology of KSHV lytic life cycle. Although they are artificial inducers, they inform that host cell signal transduction and epigenetic regulation are the mechanisms underlying the switch of the virus between latency and lytic replication. TPA is one of the most potent inducers of lytic KSHV reactivation, which is found to trigger KSHV reactivation cascade through activating protein kinase C (PKC) delta isoform, leading to stimulation of the mitogen-activated protein kinase (MAPK)/extracellular signal-regulated kinase (ERK) pathway. As a consequence of the activation of the pathway, c-Fos is accumulated and c-Jun is phosphorylated, leading to the formation of an active AP-1 complex and activation of RTA gene and the lytic cascade of KSHV (Cohen et al., 2006). Yu et al. (2007) attempted to systematically elucidate the cellular signaling that could be responsible for activating KSHV lytic cycle. They screened the effect of ectopic expression of 26,000 individual cDNA clones on RTA-dependent transcription activity in a primary effusion lymphoma (PEL) cell line latently infected with KSHV and identified a signaling molecule v-Ki-ras2 that promoted RTA transcription activity by148.1-fold. v-Ki-ras2 activates RTA and the downstream genes including PAN, kaposin, ORF57, and vIL-6. This study demonstrated that the Raf/MEK/ERK/Ets-1 pathway mediates Ras-induced activation of RTA. This pathway also mediates TPA-induced KSHV reactivation (Yu et al., 2007). The TPA and Ras-initiated signal transduction pathways that lead to activation of RTA gene is illustrated in **Figure 1**.

Ionomycin, a calcium ionophore, was found to be able to induce reactivation of KSHV from latency in PEL cells, suggesting that intracellular immobilization of calcium is able to trigger KSHV lytic replication (Chang et al., 2000). Calcium-mediated virus reactivation can be blocked by specific inhibitors of calcineurin-dependent signal transduction (cyclosporine, FK506). Furthermore, retroviral transduction with plasmid encoding VIVIT, a peptide specifically blocking calcineurin-NFAT interactions, inhibited calcium-dependent KSHV reactivation (Zoeteweij et al., 2001). Taken together, calcineurin-dependent signaling cascade induces calcium-dependent KSHV replication (reviewed in Feske et al., 2003) (**Figure 1**).

The autonomic nervous system (ANS) may also play a role in KSHV reactivation. It has been known that high levels of ANS activity accelerate the onset of AIDS-defining conditions during human immunodeficiency virus type 1 infection (Cole et al., 2003). Epinephrine and norepinephrine at physiological concentrations can induce lytic replication of KSHV in latently infected lymphoid cells via β-adrenergic activation of the cellular protein kinase A (PKA) signaling pathway. PKA increases expression of RTA and post-translational enhancement of the *trans*-activating capacity of RTA (Chang M. et al., 2005). Along with the above mentioned lytic activators, several other cellular factors, such as XBP-1, CBP, the SWI/SNF chromatin remodeling complex, the TRAP/Mediator complex, RBP-Jκ, human Notch intracellular domain, and HMGB1, have been shown to promote KSHV reactivation and/or lytic gene expression (Liang et al., 2002; Gwack et al., 2003a; Wilson et al., 2007; Yu et al., 2007; Harrison and Whitehouse, 2008; Xie et al., 2008). On the other hand, some cellular factors, such as hKFC, PARP-1, Oct-2, KAP-1, and Hey1 were found to inhibit RTA activation and viral lytic replication and may play roles in the maintenance of viral latency (Gwack et al., 2003b; Chang et al., 2009; Di Bartolo et al., 2009; Gould et al., 2009; Cheong et al., 2015).

The early finding that inhibition of the NF-κB pathway in KSHV latently infected PEL cells led to lytic gene expression and viral reactivation (Brown et al., 2003) implied a pivotal role of the NF-κB pathway in regulation latency and lytic replication. In B lymphocytes and endothelial cells, KSHV infection causes transient activation of NF-κB and sustained NF-κB activity, which is a feature of viral latency, requires expression of latent gene vFLIP (Chaudhary et al., 1999; Guasparri et al., 2004). By activating NF-κB pathway, vFLIP inhibits the expression of lytic genes through NF-κB-mediated suppression of the AP-1 pathway as well as blocking RTA transactivation in lytic gene promoters by antagonizing the RBP-Jκ coactivator (Ye et al., 2008; Izumiya et al., 2009). NF-κB is down-regulated when KSHV reactivation occurs. It was reported that lytic proteins K1, K9, and K14 inhibit NF-κB pathway (Samaniego et al., 2000; Konrad et al., 2009). Interestingly, once KSHV lytic replication takes place, some lytic proteins up-regulate NF-κB, probably for that the new increase of NF-κB is required for anti-apoptotic activities to secure the completion of viral lytic replication (Cannon and Cesarman, 2004; Martin et al., 2008).

Efficient induction of KSHV reactivation by sodium butyrate (Miller et al., 1997) and DNA demethylation agent 5′-azacytidine (Chen et al., 2001) suggests that epigenetic regulation plays an important role in the switch of the virus between latent and lytic replication. Sodium butyrate is known to be an inhibitor of histone deacetylation (HDAC) activity and treatment of cells results in histone hyperacetylation (Candido et al., 1978). Sodium butyrate activates the RTA promoter by induction of histone H3 and H4 hyperacetylation that rapidly associates chromosome remodeling protein Ini1/Snf5 with the RTA promoter (Lu et al., 2003). The recent advances in the epigenetic regulation of KSHV reactivation will be discussed as a specific topic below.

### Viral Co-infection

There are many instances where KSHV reactivation can be induced by co-infection with other viruses, such as HIV, herpes simplex virus type 1 (HSV-1), HSV-2, human cytomegalovirus (HCMV), human herpesvirus-6 (HHV-6), HHV-7, and papillomavirus (Vieira et al., 2001; Tang et al., 2012). One of the mechanisms for other viral co-infection to trigger KSHV reactivation is the secretion of inflammatory cytokines, such as oncostatin M (OSM), hepatocyte growth factor (HGF), interferon-γ (IFN-γ), and Toll-like receptors 7 and 8 (TLR7/8) (Mercader et al., 2000; Gregory et al., 2009) triggered by co-infected viruses. In contrast, KSHV lytic replication is inhibited by co-infection with Epstein–Barr virus (EBV) (Jiang et al., 2008).

It has been long suspected that HIV infection has great impacts on KSHV replication and pathogenicity. The overall risk of KS in AIDS patients was estimated to be more than 20,000-times greater than that of the general population and 300-times that of other immunosuppressed patients. AIDS-related immunosuppression appears to be a key facet to KS development and progression. HIV infection causes decreased counts of CD4+ cells and expansion of CD14^++^CD16^+^ monocytes. The unbalanced population of CD4+ cells and chronic inflammation caused by HIV leads to KSHV lytic reactivation. KSHV DNA increases in KSHV-positive patients as CD4+ count drops (Whitby et al., 1995; Martin et al., 1998; Min and Katzenstein, 1999; Keller et al., 2001; McCune, 2001; Tedeschi et al., 2001; Lukac and Yuan, 2007). T-cell proliferative responses and neutralizing antibodies to KSHV are also lower among these patients (Strickler et al., 1999; Kimball et al., 2004). The advent of HAART has resulted in recent declines of KS in the USA and many other populations (Jones et al., 2000; Mocroft et al., 2004; Iroezindu, 2016). HAART therapy leads to restoration of CD4+ cells (Lebbe et al., 1998; Jones et al., 2000; Cattelan et al., 2001; Pellet et al., 2001; Tam et al., 2002; Wilkinson et al., 2002; Nasti et al., 2003; Casper et al., 2004b; Mocroft et al., 2004), CD8+ cells (Wilkinson et al., 2002; Bourboulia et al., 2004), and NK cells (Sirianni et al., 2002). During recovery of CD4+ cells, some patients show KSHV inflammatory disease known as immune reconstitution inflammatory syndrome-KS (IRIS-KS) (Bower et al., 2005). KSHV inflammatory cytokine syndrome (KICS) is another complication of KSHV caused mainly by high levels of viral interleukin-6 (vIL6) or human IL-6 (hIL-6) and high viral load has been found in both HIV-infected and non-HIV-infected KSHV patients (Uldrick et al., 2010).

Immunosuppression clearly has an important role in KS replication and KS development, but cannot explain why the incidence of KS is highest in patients with AIDS as compare to non-AIDS immunosuppressed individuals. Onset of KS in HIV-positive individuals often occurs before appearance of severe immunosuppression. Even during HARRT therapy, rapid regression of KS was observed in patients under HARRT therapy before the complete restoration of the immune system (Zhu et al., 2014). In order to answer these questions, studies have been carried out on the direct action of HIV-1 on KSHV replication and KS pathogenicity. In a KSHV-infected primary effusion lymphoma (BC-3) cell line, infection of HIV-1 led to reactivation of the KSHV genome (Merat et al., 2002). HIV-1 was found to induce KSHV lytic replication through the activation of RTA in bystander cells by secretion of soluble factor(s) (Varthakavi et al., 1999, 2002). HIV-1 does not infect endothelial cells but it can send signaling molecules that penetrate the endothelial cells. For example, HIV-1 Tat acts as a growth factor for KS-derived endothelial cells (Guo et al., 2004; Zhu et al., 2014). It has been demonstrated that Tat triggers KSHV reactivation from latency (Harrington et al., 1997; Zeng et al., 2007) and also accelerates tumor progression induced by KSHV-encoded oncoproteins such as G protein-coupled receptor and kaposin A. In addition to Tat, accessory negative factor (Nef) is another early HIV-1-encoded regulatory protein that is an enforcing factor in the pathogenesis of HIV/AIDS and it can penetrate KSHV vIL-6-expressing endothelial cells. Both Nef and vIL-6 are internalized and in endothelial and fibroblast cells Nef synergizes with vIL-6 to promote vascular tube formation and cell proliferation. It was also shown that Nef can promote angiogenesis and vascular tube formation by activating the AKT pathway in a chicken chorioallantoic membrane (CAM) model, as well as in nude mice (Zhu et al., 2014).

Not only does HIV increases the risk of KS but co-infection of KSHV also increase the replication of HIV, moreover the entry of HIV-1 to non-infected cells. KSHV RTA, LANA, ORF45, vFLIP, ORF57 modulate the activity of the long terminal repeat region (LTR) of HIV-1 through their cooperative action with HIV-1 Tat or NF-κB signaling pathway (Caselli et al., 2001; Huang et al., 2001; Hyun et al., 2001; Sun et al., 2005; Thakker and Verma, 2016). ORF45 stimulates the transcriptional activation of HIV-1 LTR via RSK2 signaling (Karijolich et al., 2014). LANA can inhibit HIV-1-LTR via negative effect on NF-kB signaling or by induction of Staf-50 transcription factor, an inhibitor of HIV-1-LTR (Renne et al., 2001). These data show that the co-infection of KSHV can enhance or inhibit HIV-1, or vice-versa.

### Hypoxia

Clinical observation that KS tumors often appear on the extremities of the body such as feet and arm, suggests that hypoxia may contribute to KSHV reactivation and tumorigenesis. This notion has been supported by the experimental results that hypoxia induces KSHV lytic replication in PEL cells (Davis et al., 2001). These studies found that hypoxia induced accumulation of hypoxia inducible factors (HIF) 1/2. HIF-1 is a nuclear protein that activates gene transcription specifically in response to reduced cellular O_2_ concentration. HIF-1 is a heterodimer composed of HIF-1α and HIF-1β subunits, members of the basic helix–loop–helix superfamily of transcription factors. HIF-1 activity is regulated primarily by oxygen-dependent modulation of steady-state HIF-1α or HIF-2α protein levels (Semenza, 1998). In the KSHV genome, promoters of RTA and ORF34-37 lytic genes cluster contain functional hypoxia response elements (HREs) (Cai et al., 2006; Haque et al., 2006). The RTA promoter mainly responds to HIF-2α while the ORF34 promoter responds to both HIF-1α and HIF-2α, resulting in lytic reactivation of KSHV. Hypoxia/HIF-mediated KSHV lytic replication is regulated by both viral and cellular proteins. LANA is found to cooperate with HIF-1α to activate the RTA promoter. LANA also recruits chromatin remodeler KAP1 to the RTA promoter to block HIF-1α-mediated lytic replication (Cai et al., 2006, 2013). Dissociation of KAP1 from the LANA-RBP-Jκ-HIF1α complex on the promoter under hypoxic stress or knock down of KAP1 expression enhances viral reactivation (Cai et al., 2013; Zhang et al., 2014). The X-box binding protein 1 (XBP1) synergizes with HIF-1α to transacitvate the RTA promoter (Ramirez et al., 2004; Griffin et al., 2009). In addition, HIFs are able to promote aerobic glycolysis to obtain energy (ATP) and biosynthetic intermediates to sustain the growth of cancer cells, which is known as Warburg effect. Such phenomena have been found in KSHV-infected endothelial cells where KSHV up-regulates HIF-1 metabolic effector, pyruvate kinase 2 (PKM2), to maintain aerobic glycolysis in infected cells. Furthermore, as a coactivator of HIF-1, PKM2 positively regulates KS angiogenesis phenotype and increases the expression of HIF-1-dependent angiogenic factors including VEGF (Tao et al., 2015).

### Oxidative Stress

Molecules containing oxygen with unpaired electrons are known as reactive oxygen species (ROS). These unpaired electrons of oxygen can react to form partially reduced, highly reactive species, for example, superoxide (O_2_), hydrogen peroxide (H_2_O_2_), hydroxyl radicals, and peroxynitrites (Fruehauf and Meyskens, 2007). ROS originate from various cellular enzyme systems, such as the mitochondrial electron transport chain, the NADPH oxidase complex, xanthine oxidase, lipoxygenase, cyclooxygenase, and peroxisomes. Increasing evidence suggests the role of oxidative, nitritive stress, and ROS in KSHV reactivation in KS patients (Gil et al., 2003; Mallery et al., 2004; Ye et al., 2011). During infections and inflammatory responses, host phagocytes produce and release excessive amounts of H_2_O_2_, which induces expression of RTA, ORF57, ORF59, K8, ORF65 in a dose-dependent manner in BCBL1 and human umbilical vein endothelial cells (HUVEC) latently infected with KSHV (Ye et al., 2011). H_2_O_2_ induction of KSHV reactivation depends on the activation of MAPK ERK1/2, JNK, and p38 pathways (Li et al., 2011; Ye et al., 2011) (**Figure 1**). Cells express multiple antioxidant enzymes such as catalase and glutathione peroxidase to remove H_2_O_2_. The cells with catalase knock down by siRNA increased the expression of KSHV lytic transcripts. Disruption of the intracellular redox balance through depletion of the antioxidant glutathione or inhibition of the antioxidant enzyme catalase also induces KSHV reactivation, confirming that hydrogen peroxide induces reactivation.

## Mechanisms that Regulate the Switch of KSHV Between Latency and Lytic Replication

### Kinetic Classification of KSHV Lytic Genes

Like all herpesviruses, KSHV lytic replication proceeds as viral lytic genes are induced in a cascade fashion. The kinetics of KSHV gene expression has been studied in both tissue culture and clinical samples. Miller’s group classified KSHV genes in accordance to their response to treatment of cycloheximide (CHX) – a protein synthesis inhibitor and phosphonoacetic acid (PAA) – an inhibitor of DNA polymerase (Sun et al., 1999). The genes that constitutively express in both latent and lytic life cycles are designated as latent genes. The genes that express only after induction of cells with chemical inducers such as TPA or butyrate are lytic genes. Among them, the genes that can be induced by TPA or butyrate in the presence of CHX are termed IE genes, as their induced expression does not require *de novo* protein synthesis. DE genes express after IE gene products are synthesized but prior to viral DNA synthesis. Their expression is blocked in the presence of CHX but not affected by inhibition of viral DNA replication by PAA. Late (L) gene expression is coupled with the viral lytic replication and can be only detected after viral DNA replication initiation. In accordance with these criteria, a class of genes including RTA, K8, and ORF45 are designated IE genes (Zhu et al., 1999). DE genes, in general, encode enzymes and regulatory proteins required for supporting viral DNA replication and creating favorable cellular environment for success of lytic viral replication. The majority of the genes for viral structural proteins, including capsid, tegument, and glycoproteins are late genes with exceptions, that is, if they carry out other functions in other dynamical phases of viral life cycle (Saveliev et al., 2002). How the expression of KSHV genes in different kinetic categories is regulated in coordination is the central question on KSHV switch between latent and lytic cycle and proceeding of lytic replication.

### Immediate-Early Gene Product RTA Is the Major Viral Lytic Switch Protein

Among KSHV IE gene products, ORF50-encoded RTA is a key regulator for the switch between latent and lytic viral life cycle. RTA expression is necessary and sufficient for KSHV reactivation. Ectopic expression of RTA in latently infected B-lymphocytes results in completion of the cascade leading to KSHV lytic replication (Lukac et al., 1998; Sun et al., 1998). RTA is conserved among all members of gamma-herpesvirus family such as EBV, rhesus rhadinovirus (RRV), herpesvirus saimiri (HVS), murine herpesvirus 68 (MHV-68), bovine herpesvirus 4, and equine herpesvirus 2, but only the RTAs from gamma-2-herpesviruses (KSHV, HVS, and MHV-68) acts as a lytic replication activator (Sun et al., 1998; Wu et al., 2000; Goodwin et al., 2001). RTA is a transcription activator with unique features: its DNA binding region does not contain any well-characterized DNA binding motifs and the overall protein shows no homology to any known cellular transcriptional activators. Therefore, RTA could be an attractive target for antiviral therapy.

**FIGURE 2:**
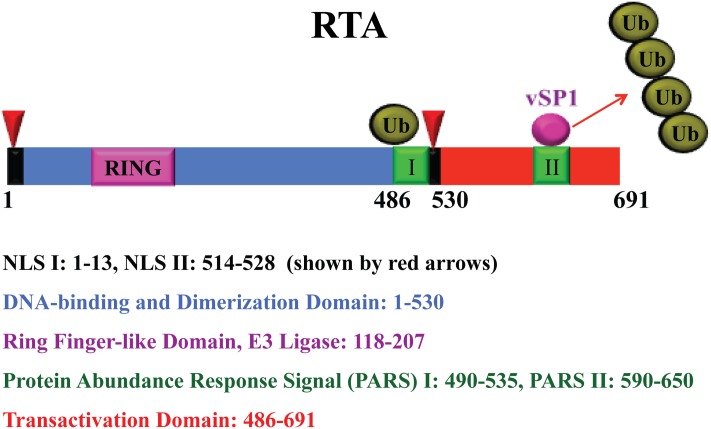
**Schematic representation of RTA and functional domains.** The RTA nuclear localization signals (NLS), RING finger-like domain, protein abundant regulatory signal (PARS), and transactivation domain are shown with color codes as indicated. RTA undergoes auto-ubiquitylation by the E3 ligase activity of the ring finger-like domain for a self-control of its abundance. A microPeptide (vSP1) interacts with PARS II domain and lifts the abundance restriction by blocking RTA self-ubiquitylation.

RTA is a 691 amino acid protein (**Figure 2**). As a nuclear protein, it has two arginine- and lysine-rich nuclear localization signals, one at the N-terminus in aa 1-13 and the other near its C-terminus in aa 514-528 (West and Wood, 2003; Bu et al., 2008). The DNA binding and dimerization domains are located at the aa 1-530 (Lukac et al., 2001; Chang and Miller, 2004). The transactivation domain has been defined at the aa 486-691, which is highly acidic and contains numerous charged amino acids (Lukac et al., 1999; Seaman et al., 1999) (**Figure 2**). This region contains four repeated units of a highly hydrophobic domain, known as activation domains 1–4 (AD1–AD4), with sequence homology to other transcriptional factors such as VP16 domain A (Lukac et al., 1999). Deletion of acidic activation domain results in dominant negative of RTA and inhibits viral lytic reactivation. RTA also has proline-rich region, serine/threonine-rich region, cysteine/histidine-rich region, leucine heptapeptide repeat domain, and 4-hydrophobic-acidic repeat regions (486–691) (Guito and Lukac, 2015). A region (aa 520-535) centered with a basic motif (KKRK) regulates abundance of RTA. This region (aa 520-535), together with a downstream sequence (aa 590-650), constitutes the protein abundance response signal (PARS). Mutants or deletion of these motifs greatly enhanced the abundance of RTA in cells as well as DNA binding activity. Interestingly the PARS mutations inhibit RTA transactivation activity, suggesting that this motif is required for controlling RTA abundance, DNA binding and transactivation activity (Chang and Miller, 2004; Chang et al., 2008). Given that the PARS mutations gave rise to an RTA variant with faster electrophoretic mobility (form B of RTA), PARS-mediated regulations (abundance, DNA binding and transactivation) may involve post-translational modification (PTM).

RTA activates a number of viral promoters by different mechanisms as follows: (1) RTA binds to DNA of three promoters for PAN RNA, ori-Lyt-associated RNA (T1.4) and T0.7, directly and tightly by recognizing the RTA responsive elements (RREs). These three RREs share a 16-bp core sequence and designated type I RREs (Lukac et al., 2001; Song et al., 2002; Wang et al., 2003a); (2) RTA activates the promoters of ORF57 and K8 (delayed-early promoter) by binding to type II RREs, which exhibit varying binding affinity for RTA but required cooperative interaction with various cellular factors (Liang and Ganem, 2003; Wang et al., 2003a,b; Wang S.E. et al., 2004; Wen et al., 2009). Transactivation of ORF57 and K8 genes by RTA requires a direct interaction with RBP-Jκ and RTA may bind to the type II RREs (contain RBP-Jκ binding sites) by piggybacking on to the cellular protein (Liang et al., 2002; Wang and Yuan, 2007). In fact, RBP-Jκ binding has been identified to at least 99 sites within the KSHV genome and involves as many as 34 RTA-activated viral genes, which suggests the potential for an RTA-RBP-Jκ complex to induce the entire lytic cascade (Guito and Lukac, 2015). KSHV may regulate cellular genes using RTA-RBP-Jκ complex as a ChIP-seq analysis shows 3,846 Notch1 binding sites and 2112 of RBP-Jκ binding sites in human T-lymphoblastic leukemia cell lines (Wang et al., 2011). For example, RTA is able to activate cellular genes for surface protein CD21 and CD23 through interaction with RBP-Jκ (Chang H. et al., 2005). RTA was also found to activate its target genes in combination with other cellular transcription factors, like Octamer-1, C/EBPα, C-Jun, SP1, and STAT3 (Sakakibara et al., 2001; Wang et al., 2003a, Wang S.E. et al., 2004; Carroll et al., 2006, 2007; King et al., 2015); (3) RTA has been also found to promote gene expression through the degradation of transcriptional repressors. RTA exhibits an ubiquitin E3 ligase activity that directs proteasome-mediated degradation of lytic gene expression repressor including Hey-1 and LANA (Yang et al., 2008; Gould et al., 2009; reviewed in Guito and Lukac, 2012). Through these various mechanisms, RTA efficiently activates gene expression cascade leading to completion of viral lytic replication. These all have been illustrated in **Figure 1**.

### Regulation of RTA Function by LANA

As RTA is an important regulator controlling the switch between latency and lytic viral replication and determining the fates of both virus and host, it has to be under control with maximum security. RTA is the first viral gene to be expressed following *de novo* infection. Expressed RTA immediately activates LANA gene, thereafter initiating the establishment of latency (Lan et al., 2005b). LANA expression in turn down-regulates RTA expression (Lan et al., 2004). In the cell with a LANA-null recombinant mutant KSHV, there is tremendously increased expression of lytic genes including RTA, vIL-6, ORF57, ORF59, and K8.1, indicating LANA represses expression of RTA as well as other RTA-responsive genes (Lu et al., 2006; Li et al., 2008). LANA interacts with the RTA promoter and inhibits RTA gene expression. LANA-mediated repression of RTA promoter activity depends on RBP-Jκ binding sites. LANA recruits RBP-Jκ protein to the RTA promoter and blocks the self-activation of the promoter by competing with RTA for RBP-Jκ-binding (Lan et al., 2005a). Therefore, the switch of KSHV between latency and lytic cycle is controlled by the interplay between LANA and RTA proteins in infected *cells*.

### Regulation of RTA Abundance by Self-Ubiquitylation and microPeptides

Another level of regulation is on the protein abundance of RTA. Miller et al. (1997) reported that the level of RTA in cells is controlled by the regulatory domains called PARS, which consists of two components, namely PARS I (aa 490-535) and PARS II (aa 590-650) (Chang et al., 2008). Mutation or deletion of either component results in abundant expression of RTA protein. The N-terminus of RTA contains a RING finger-like domain, which possesses E3 ligase activity that ubiquitylates and degrades some viral and cellular targets, including K-bZIP, PML, IRF-7, and K-RBP (Yu et al., 2005; Yang et al., 2008; Izumiya et al., 2013; Ehrlich et al., 2014). Our preliminary data showed that the E3 domain of RTA is also capable of self-ubiquitylating RTA itself possibly at the PARS domain. Therefore, RTA utilizes the self-ubiquitylation mechanism to maintain RTA at a relatively low level in order to prevent the virus from unwanted reactivation.

Interestingly, KSHV has also developed a mechanism to counteract the self-ubiquitylation-mediated abundance control using a virally encoded microPeptide. When KSHV switches to lytic life cycle, elevated RTA promotes the transcription from the strand opposite of the ORF50 DNA that gives rise to a previously annotated non-coding RNA, termed T3.0 (Saveliev et al., 2002). Originally, it was found that T3.0 RNA could relieve the PARS-mediated RTA abundance restraint and increase the RTA level in cells. Further investigation indicated that the up-regulation of RTA expression is not mediated by the non-coding RNA, but by a microPeptide translated from T3.0 RNA. Although T3.0 was annotated as a non-coding RNA, T3.0 transcript carries a series of small open reading frames (sORF). Ganem’s group reported that the T3.0 RNA is associated with ribosomes and some of the sORFs in T3.0 RNA do have the potential to make small peptides (Xu and Ganem, 2010). The translation of these sORFs in cells was confirmed and two small peptides (vSP-1 and vSP-2) were identified that are translated from potential sORFs encompassing the nucleotides 74356-74035 and 74029-73912 within T3.0 RNA. These two small peptides, 48- and 27-aa in length, respectively, are designated as microPeptides (vSP-1 and vSP-2). Two lines of experiment were carried out to determine if these two microPeptides are capable of regulating RTA. First, the initiation codons of vSP-1 and vSP-2 were altered from AUG to AAG. When the mutants were introduced into cells along with an RTA expression vector, the mutation at vSP-1 initiation codon abolished the RTA enhancement activity of T3.0, while the mutant bearing the mutation at the vSP-2 initial codon retained the activity comparable to wild-type T3.0. Second, the ectopic expression of vSP-1 led to the increase of RTA abundance similar to that of T3.0 RNA. These results strongly suggested that T3.0 could regulate RTA expression through a microPeptide (Jaber and Yuan, 2013).

The microPeptide vSP-1 physically interacts with RTA at the PARS II domain. The interaction blocks the self-ubiquitylation of RTA by the internal E3 ligase located at the RING domain (Jaber and Yuan, 2013) (**Figure 2**). Thus the balance of RTA self-ubiquitylation and the microPeptide action provide a precise control of RTA abundance and activity.

### Role of Pin1 Isomerase in Regulation of KSHV Lytic Reactivation

Most of amino acids exist in *trans*-form in proteins except proline which is known as imino acid as it possesses both *cis*- and *trans*-isoforms. This *cis*/*trans*-isomerization plays an important role in protein folding. Peptidyl-prolyl *cis*/*trans* isomerase Pin1 is an enzyme catalyzing prolyl hydroxylation and prolyl isomerization (Wedemeyer et al., 2002). Pin1 targets phosphoserine or phosphothreonine–proline motifs and convert them from *trans-* to *cis*-form, a post-phosphorylation event. The locking mechanism of Pin1 controls the timing and amplitude of a specific process such as protein folding or G1/S check point (Lu et al., 2007). Dysregulation of Pin-1 has been associated with cancers and Alzheimer’s disease (reviewed in Guito and Lukac, 2015).

In RTA, prolines constitute 17% conserved amino acids among members of the *gamma-Herpesviridae* subfamily and these prolines are present in critical domains like oligomerization, DNA binding, and RBP-Jκ binding domain, suggesting an important role of these prolines in RTA function (Bu et al., 2007; Guito et al., 2014). RTA contains 15 potential binding and regulatory motifs for Pin1 and RTA indeed physically interacts with Pin1. RTA transactivation is enhanced by Pin1 at two delayed-early viral promoters for PAN RNA and ORF57. However, ectopic expression of Pin1 inhibits late gene expression and production of infectious virus (Guito et al., 2014; Guito and Lukac, 2015). These data suggest that Pin1 may act as KSHV lytic cycle timer that controls the balance between viral lytic replication and host cell lysis through regulating RTA expression and downstream activity.

## Epigenetic Regulation of KSHV Reactivation

### Chromosome Conformation and Chromatin Remodeling

**FIGURE 3:**
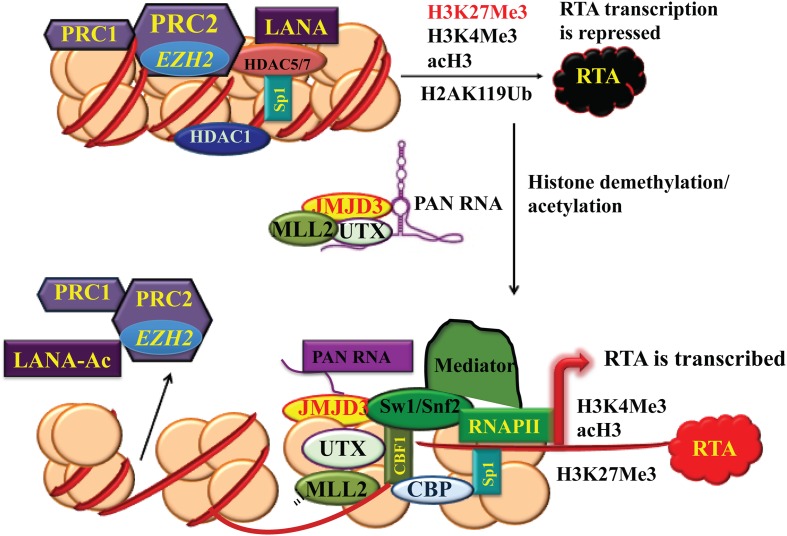
**Chromatin remodeling of KSHV RTA promoter region during latency and lytic reactivation.** During latency, the chromatin of the immediate-early (IE) promoter region contains bivalent histone marks including both activating acH3, H3K4me3 and repressive H3K27me3. Polycomb repressive complex 2 (PRC2) colocalizes with H3K27me3 on RTA and PRC1 complex generates H2AK119Ub2 to repress RTA transcription. When the virus enters lytic life cycle, PRC2 dissociates from the genomic regions of IE and delayed-early (DE) genes. PAN RNA recruits histone demethylases UTX and JMJD3 and histone-lysine *N*-methtltransferase 2D (KMT2D or MLL2) to the chromatin. As a result, the decrease in H3K27me3 and increase in H3K4me3 and acH3 results in activation of the IE and DE promoters and lytic gene expression.

First implication that KSHV is epigenetically controlled comes from its activation by histone deacetylase (HDAC) inhibitors sodium butyrate or trichostatin A, and DNA methyltransferase inhibitor 5-Azacytidine (5-AzaC) (Renne et al., 1996; Lu et al., 2003). These chemicals have global effects on chromatin modeling and gene expression. Epigenetic alterations involve many enzymes for histone modifications and chromatin modeling. Histone modification enzymes can be classified into three categories: (1) writers: histone acetyltransferases (HATs) and histone methyltransferases (HMTs); (2) erasers: HDACs and histone lysine demethylases (KDMs and Jumonji families); and (3) readers: proteins that recognize these histone modifications. The KSHV genome is histone-free in virions but quickly adopts a highly organized chromatin structure following *de novo* infection. The chromatinated KSHV genome is distinctively enriched with the activating (acetylated H3 and H3K4me3) and repressive (H3K9me3 and H3K27me3) histone modifications. During latency, the latency-associated gene cluster is associated with activating histone marks accompanied by transcriptionally active RNA polymerase II binding. The promoters of RTA and some DE genes have bivalent chromatin marks (enriched with both activating AcH3, H3K4me3 and repressing H3K27me3 histone modifications), whereas the late genes are in repressive H3K9me3- and H3K27me3-marked heterochromatin (Gunther and Grundhoff, 2010; Toth et al., 2010; Uppal et al., 2015). Polycomb repressive complex 2 (PRC2), composed of subunits EZH2, SUZ12, EED, and RbaAp48/46 (Simon and Kingston, 2009), colocalizes with the H3K27me3 mark on the entire KSHV genome and EZH2 catalyzes the methylation of H3K27 during latency (Toth et al., 2010). In addition, JMJD2A, a H3K9me3 demethylase, is associated with the latent region and prevents the methylation of H3K9 (Chang et al., 2011). Along with PRC2 complex, PRC1 complex that mono-ubiquitinates lysine 119 of H2A (H2AK119ub) and represses transcription has been also detected on the RTA promoter (Toth et al., 2013). Once viral lytic cycle is induced, EZH2 of PRC2 dissociates from the genomic regions of IE and DE genes concurrent with decreasing H3K27me3 and increasing acH3 and H3K4me3 with subsequent lytic gene expression (**Figure 3**). This shift allows RTA to recruit cellular transcriptional cofactors such as TBP, CBP, Brg1, BAF170, TRAP220, and TRAP100, leading to optimal RTA production and activation of entire viral lytic program (**Figure 3**) (Gunther and Grundhoff, 2010; Toth et al., 2010).

Interestingly, KSHV non-coding RNA PAN was found to play a critical role in the changes in the histone modification that leads to shift of the KSHV IE and DE gene region to activating chromatin. A PAN-null recombinant mutant KSHV fails to express RTA and to enter lytic cycle. PAN RNA, serving as a guide RNA, recruits histone demethylases UTX and JMJD3 to the IE and DE gene location to remove the repressive marks H3K27me3 in the RTA promoter. PAN RNA also delivers H3K4me3 methyltransferase MLL2 (KMT2D) to the region to add the reactivating marks (Rossetto and Pari, 2012, 2014) (**Figure 3**).

The chromatin remodeling is region specific with certain histone marks enriched only on specific viral genomic regions. Increasing data suggest that chromosome conformation may determine the epigenetic patterning of a viral genome. The chromatin organizing factor CTCF has been implicated in the control latent and lytic gene expression of KSHV and other herpesviruses. CTCF can prevent the spread of activating and repressive chromatin from one regulatory domain to another by acting as an insulator (reviewed in Lieberman, 2013). In addition, CTCF has been found to colocalize with cohesin at several sites throughout the KSHV genome. During latency, the major CTCF-cohesin peak resides in the first intron of the latent transcription (LANA-vCyclin-vFLIP) region and the CTCF binding site has been shown to be important for KSHV episome stability, maintenance and efficiency of *de novo* infection (Stedman et al., 2008). Using chromosome conformation capture technique, Kang et al. (2011) found that CTCF-cohesin binding sites are involved in DNA-loop formation with other regions. CTCF-cohesin mediates a loop between the latent transcription unit and the RTA promoter region. Depletion of cohesin subunits leads to robust reactivation of lytic gene expression (Kang et al., 2011; Li et al., 2014), suggesting that the CTCF-cohesin complex at the latency control region functions as a repressor of lytic transcription. Furthermore, CTCF, together with cohensin, protects the lytic control region and retain Pol II at the lytic promoter in a conformation poised for rapid response to reactivation signals (Chen et al., 2012). Taken together, the KSHV genome is organized into chromatin loop mediated by CTCF-cohesin interaction and the dynamic linkages between regions of the viral genome insulate and coordinate latent and lytic gene expression.

### Regulation of Reactivation at RNA Level by microRNAs

Viruses encode microRNAs. Similar to cellular microRNAs, viral miRNAs target host immune system, cell cycle regulation, cell apoptosis, and are responsible for viral pathogenesis or oncogenesis. Twelve KSHV pre-miRNAs (miR-K1∼miR-K12), encoding 25 mature miRNAs, were identified within the KSHV genome (Cai et al., 2005; Samols et al., 2005). These miRNA genes are clustered in the latency-associated region and highly expressed in latently infected primary effusion lymphoma cells (Gottwein and Cullen, 2008). Substantial evidence supports a role of some viral miRNAs in maintenance of viral latency, either directly or indirectly. A recombinant KSHV with a deletion of miR-K1-11 (ΔmiR) was found to consistently express higher levels of RTA mRNA than wild-type virus (Lu F. et al., 2010). In particular, miR-K9 targets the 3′UTR of RTA mRNA and suppresses RTA and lytic reactivation (Bellare and Ganem, 2009; Lin and Ganem, 2011). miR-K3 indirectly represses RTA by targeting nuclear factor I/B (NFIB, a transcription factor) that activates RTA promoter (Lu C.C. et al., 2010) (**Figure 1**). Knockdown of KSHV miR-K3 and miR-K11 increased expression of lytic genes as well as virus production from latently infected PEL cells. These two viral miRNAs contribute to maintenance of latency by decreasing RTA expression indirectly, presumably via down-regulation of MYB, C/EBPα, and Ets-1, and possibly other host transcription factors (Plaisance-Bonstaff et al., 2014).

Kaposi’s sarcoma-associated herpesvirus-encoded miRNAs were also found to influence epigenetic regulation of viral latency. Comparison of epigenetic marks in ΔmiR-KSHV revealed decreases in H3K9me and increases in AcH3 as well as a striking loss of DNA methylation throughout the viral genome (Lu F. et al., 2010). miR-K4-5p was found to target retinoblastoma (Rb)-like protein 2 (Rbl2), which is a known repressor of DNA methyl transferase 3a and 3b mRNA transcription (Lu C.C. et al., 2010). Thus, KSHV miRNA targets multiple pathways to maintain the latent state of the KSHV genome, including repression of Rta and regulation of epigenetic reprogramming.

Several KSHV miRNAs, miR-K3, miR-K10a, and miR-K11, act as viral analogs of the human cellular miRNAs miR-155, miR-142-3p, and miR-23, respectively. These cellular and viral miRNAs share the repertoire of targeted genes (Gottwein et al., 2007, 2011; Skalsky et al., 2007; Manzano et al., 2013). KSHV miR-K10 shares the same seed sequence with cellular miR-142-3p and targets TGF-β type II receptor in PEL and KSHV-infected endothelial cells (Lei et al., 2012). KSHV miR-K1 was reported to directly target and repress of cellular protein IκBα, the primary inhibitor of NF-κB (Lei et al., 2010). By repressing IκBα, miR-K1 activates NF-κB/IL-6/STAT3 signaling and functions as an oncogene (Chen et al., 2016).

## Regulation of Lytic DNA Replication

The cascade of IE and DE gene expression leads to lytic DNA replication. KSHV lytic DNA replication initiates at an origin (*ori-Lyt*) and requires *trans*-acting elements, both viral and cellular. Unlike viral latent DNA replication that initiates at *ori-P* and proceeds bi-directionally, the lytic replication initiates from a distinct origin (*ori-Lyt*) and proceeds via a rolling-circle fashion. Unlike viral latent DNA replication that depends on host cellular DNA polymerase and accessory factors, the lytic replication utilizes its own DNA polymerase and other factors. Unlike viral latent DNA replication that is in synchrony with the host cell to maintain a stable number of viral DNA episomes in each cell, the lytic DNA is amplified hundreds-fold during lytic replication. Through a rolling-circle mechansim, viral lytic DNA replication results in the generation of concatemeric genomic DNA that is subsequently cleaved into 170 kb long genomes during packaging.

### Origins of Lytic DNA Replication

**FIGURE 4:**
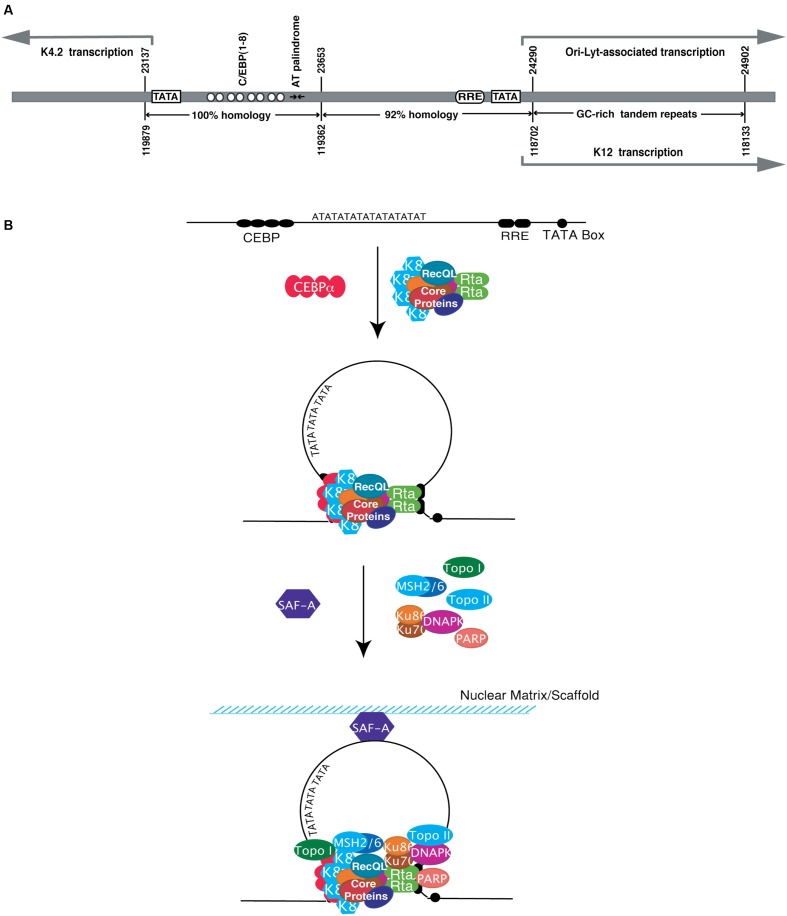
**Structure of the KSHV origin of DNA replication (*ori-Lyt*) and formation of viral replication initiation complex formation.** **(A)** *Ori-Lyt-R* and *ori-Lyt-L* are superimposed to show their commonalities. The positions of various characteristic motifs (TATA boxes, C/EBP binding motifs, AT palindrome, RRE and GC tandem repeats) are as indicated. The homologies of subregions between two *ori-Lyts* are compared and shown on the bottom. **(B)** Model for formation of pre-replication complex and replication initiation complex on KSHV *ori-Lyt.* Six core replication proteins form pre-replication complex. The pre-replication complex is then loaded at a KSHV *ori-Lyt* by a two-point-contact through RTA and K8, each of which interacts with their binding motifs in the *ori-Lyt*. The interaction may lead to looping and distortion of the *ori-Lyt* DNA. Furthermore, some cellular proteins are also recruited to the complexes. RecQL is likely to be a component of pre-replication complex and recruited to *ori-Lyt* together with viral core replication proteins in the complex through K8 and RTA. The loading of the pre-replication complex on *ori-Lyt* may cause structural changes of *ori-Lyt* DNA that facilitates the recruiting of more cellular proteins, including MSH2/6 and DNA-PK/Ku86/70, to the *ori-Lyt*. Scaffold attachment factor A (SAF-A) binds directly to *ori-Lyt* DNA and may tether the *ori-Lyt* DNA to the nuclear scaffold or matrix for efficient DNA replication (Modified from Wang Y. et al., 2004).

Two duplicated copies of lytic DNA replication origin [referred to as *ori-Lyt* (L) and *ori-Lyt* (R)] were identified in the KSHV genome, located between open reading frames K4.2 and K5 and between K12 and ORF71, respectively (AuCoin et al., 2002; Lin et al., 2003). These two *ori-Lyts* share an almost identical 1.1 kb core component sequence, followed by a 600 bp GC-rich repeats that are represented as 20 and 30 bp tandem arrays (Lin et al., 2003). Each 1.7 kb *ori-Lyt* sequence is necessary and sufficient as a *cis*-acting signal for KSHV lytic DNA replication (Lin et al., 2003). Four motifs and regions within the 1.7 kb *ori-Lyt* sequence were identified to be essential for the initiation of DNA replication. First, an 18-bp AT-palindromic sequence is seen in both *ori-Lyt* (L) and *ori-Lyt* (R) (**Figure 4A**) and deletion of the palindrome or introduction of GC pairs in the sequence abolished the DNA replication (Wang et al., 2004). Such an AT-rich palindrome sequence is believed to facilitate DNA unwinding and enhance helicase activity during DNA replication (Challberg and Kelly, 1989; DePamphilis, 1993). Second, eight C/EBP binding motifs that are organized as four spaced C/EBP palindromic pairs within a 240-bp sequence were found to be important for viral lytic replication (**Figure 4A**) (Wu et al., 2003b; Wang Y. et al., 2004). Each palindrome contains two head-to-head CCAAT consensus motifs that are separated by a 13- or 12-bp spacer sequence. C/EBPα is found to bind to some of the C/EBP palindromes and the viral protein K8/KbZIP associates with *ori-Lyt*, likely through piggybacking on the C/EBPα (Wang Y. et al., 2004; Wang et al., 2006). Third, an RRE adjacent to a TATA box was found at the end of 1.1 kb *ori-Lyt* core element (near the 600 bp GC-rich repeats) (**Figure 4A**). This motif functions as a transcriptional promoter that directs a transcription of a 1.4 kb RNA containing GC-rich repeats in the *ori-Lyt-L*. But in *ori-Lyt-R*, the equivalent promoter controls the synthesis of K12/T0.7 RNA that also contains GC-rich repeats (Wang Y. et al., 2004). The transcription events under the control of these promoters are essential for DNA replication from these *ori-Lyts*, as premature termination of the transcription by inserting an artificial polyadenylation signal upstream of the GC-rich repeats completely abolished the DNA replication (Wang Y. et al., 2004). In addition, the RRE also plays a role in recruiting pre-replication complexes, composed of RTA, K8 and six core machinery proteins, to the *ori-Lyt* DNA (Wang et al., 2006). Forth, the 600-bp GC-rich tandem repeat sequences are also indispensable for *ori-Lyt*-dependent DNA replication (Lin et al., 2003).

The biological significance of the existence of two *ori-Lyts* in the KSHV genome is still elusive. A study with the recombinant KSHVs in which one or both of the *ori-Lyts* were removed from the viral genome demonstrated that *ori-Lyt-L* is sufficient to propagate the viral genome in Vero cells whereas *ori-Lyt-R* alone seems inert to direct amplification of viral DNA (Xu et al., 2006). It is possible that two similar but not identical *ori-Lyts* in KSHV have distinct preferences for certain cell lines or modes of infection, therefore multiple *ori-Lyts* in a viral genome may provide optimal virus fitness for different host cell types and tissues. This hypothesis was recently validated in MHV-68 where two *ori-Lyts* are differentially bound by different proteins and alteration of the expression of these proteins affected the replication of mutant viruses lacking either the left or the right *ori-Lyt* (Sattler et al., 2016).

### *Trans*-Acting Elements Required for *ori-Lyt*-Dependent DNA Replication

Two viral proteins, namely RTA and K8/K-bZip, recognize and bind to *ori-Lyt* in a sequence specific manner and are suggested to function as *ori-Lyt* binding proteins (OBPs). RTA binds to an RRE in *ori-Lyt*. RTA plays dual roles in *ori-Lyt*-dependent DNA replication. As mentioned earlier, RTA binds to the RRE within *ori-Lyt* and functions as a transcription activator to promote the transcription of *ori-Lyt*-associated transcripts (T1.4 RNA from *ori-Lyt*-L and K12/T0.7 RNA from *ori-Lyt*-R). In addition, RTA is also responsible for the recruiting of pre-replication complex to the *ori-Lyt* through its binding to the RRE and interacting with other core replication machinery proteins (Wang et al., 2006). RTA is a component of the viral pre-replication complexes composed of at least six core replication proteins, K8/K-bZIP, and RTA. RTA, together with K8/K-bZip (see below), mediates the loading of the pre-replication complex on the *ori-Lyt* DNA. Deletion or mutation of the RRE abolished the association of replication proteins with *ori-Lyt* DNA (Wang Y. et al., 2004; Wang et al., 2006).

K8/KbZIP was demonstrated to bind to *ori-Lyt* DNA as well (Lin et al., 2003). The binding site was mapped to the C/EBP palindromes in KSHV *ori-Lyts*. However, K8 does not bind to *ori-Lyt* DNA directly but through piggybacking on DNA-bound C/EBPα (**Figure 4B**) (Wu et al., 2003a,b; Wang Y. et al., 2004). A transient cotransfection-replication assay demonstrated an essential role of K8 in *ori-Lyt*-dependent DNA replication as omission of K8 expression plasmid in the co-transfection led to lack of detectable DNA replication (AuCoin et al., 2004).

Both RTA and K8 are able to interact with core replication machinery proteins and recruit these proteins to *ori-Lyt* DNA to form a pre-replication complex (Wang et al., 2006). The core replication machinery proteins include a DNA polymerase (POL), a polymerase processivity factor (PPF), a single-stranded DNA binding protein (SSB), a helicase (HEL), a primase (PRI), and a primase-associated factor (PAF). Co-expression of these proteins and the OBPs (RTA and K8/KbZIP) in eukaryotic cells is sufficient to support DNA replication of a plasmid containing a KSHV *ori-Lyt* (AuCoin et al., 2004).

### Formation of Replication Initiation Complex and Proceeding of KSHV *ori-Lyt*-Specific DNA Replication

There were two models proposed for the formation of herpesviral replication initial complex on *ori-Lyt* DNA. (1) Herpesviral lytic replication initiates with the binding of OBPs to *ori-Lyt* elements. Subsequently, the OBPs recruit DNA replication enzymes and accessory factors to the origin where viral replication complex forms. (2) Viral pre-replication complexes composed viral replication core machinery proteins and OBP(s) are formed in solution and subsequently loaded on *ori-Lyt* DNA to form viral replication complex. Experimental evidences support the latter model for KSHV *ori-Lyt*-dependent DNA replication. Both K8/K-bZip and RTA are components of a multi-protein complex composed of the six viral replication core machinery proteins (POL, PPF, SSB, HEL, PRI, and PAF). The complex, designated pre-replication complex, forms in solution regardless of the presence of the viral genomic DNA (Wu et al., 2001; Wang et al., 2006) and binds to DNA fragments with the K8 binding region (the C/EBP cluster) or RRE, respectively, suggesting K8/K-bZip and RTA can independently mediated the association of pre-replication complex with *ori-Lyt* DNA through their specific binding sites (Wang et al., 2006). These findings leads to a model for recruiting of pre-replication complexes to *ori-Lyt* by RTA and K8, which is illustrated in **Figure 4B**. The two-contact-point binding may bring the two ends of the *ori-Lyt* element together, looping the DNA between the K8 and RTA binding sites. The 18-bp AT palindrome sequence is located in the center of the loop. The looping causes bending and distortion of the *ori-Lyt* DNA, facilitating unwinding of the DNA sequence (Wang et al., 2006).

In addition to viral components, many cellular proteins are also incorporated in the replication initiation complex. They include topoisomerases (Topo) I and II, RecQL, poly(ADP-ribose) polymerase I (PARP-1), DNA-PK, Ku86/70 autoantigens, MSH2/6, and scaffold attachment factor A (SAF-A) (**Figure 4B**). RecQL appears to associate with pre-replication complexes and be recruited to *ori-Lyt* through RTA and K8. Topo I and II, PARP-1, DNA-PK, Ku86, and MSH2 were not detected in pre-replication complexes but were present in replication initiation complexes on *ori-Lyt* (Wang et al., 2008). Topo I and II have been demonstrated to be essential for viral lytic replication as depletion of Topo I and II using an shRNA knock down approach severely inhibited viral lytic DNA replication as well as virion production (Gonzalez-Molleda et al., 2012).

## Regulation of Late Genes

The genes coding for KSHV structural proteins (capsid proteins, tegument protein, envelope glycoproteins, and relevant auxiliary factors) do not express until viral lytic DNA replication begins. This class of genes is designated late (L) genes. Among all three herpesviral subfamilies (alpha-, beta- and gamma-herpesviridae), the late gene regulation share similar features (Homa et al., 1986; Johnson and Everett, 1986; Anders et al., 2007). (1) The promoters of late genes are typically very simple defined by small regions surrounding the TATA motif. For example, KSHV K8.1 promoter consists of a 24-bp sequence with a TATA-like core (AATATTAAAGGG) and is regulated in KSHV lytic replicating cells by *trans* (Tang et al., 2004). (2) Late genes expression is coupled with viral lytic DNA replication as expression of an authentic late gene can be blocked by a viral DNA polymerase inhibitor PAA. The activity of the K8.1 promoter can be enhanced (>15-fold) by a KSHV *ori-Lyt* sequence in *cis*. The promoter activity is sensitive to PAA with an inhibition up to 97%, fully imitating the activation of an authentic late gene in response to viral lytic replication (Tang et al., 2004).

The *trans*-acting elements that control KSHV late gene expression have been characterized recently. It was shown that KSHV late gene expression requires the coordination of six DE viral proteins, namely ORFs 18, 24, 30, 31, 34, and 66, which form a late gene transcription preinitiation complex (vPIC) (Brulois et al., 2015; Davis et al., 2015a). Furthermore, ORF24 is a TATA box binding protein (TBP) homolog that recruits RNA polymerase II to the late promoter for the transcription from the promoter (Davis et al., 2015b). The mechanism that couples late gene activation to viral DNA replication has not yet been elucidated. A putative model has been that the restriction of late gene activation may attribute to viral genome structure and viral DNA replication temporally remodels the chromatin structure, therefore relieving the restrain for the late gene expression and allowing the vPIC binds to the TATA box region of late gene promoter.

## Assembly of Viral Particles and Release of Infectious Virions

Lytic replication of viral genomic DNA and synthesis of viral lytic proteins leads the cascade to the next phase of KSHV lytic replication – viral particle assembly and virion release. Assembly of viral particle is autocatalytic and intrinsic information governing the assembly process is borne in the viral structure proteins. However, some cellular protein and machineries are harnessed or usurped by virus to assist viral particle transport and membrane budding.

**FIGURE 5:**
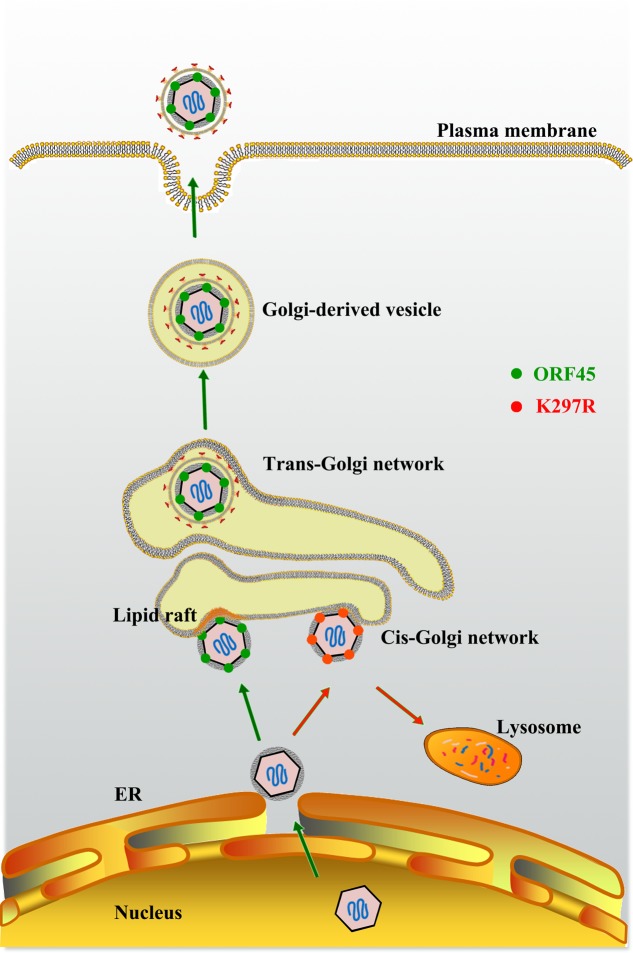
**Model for role of ORF45 in lipid raft (LR)-localization and KSHV final envelopment.** After budding into the cytoplasm, nucleocapsids gain tegument in the cytoplasm. Tegument protein ORF45 directs tegumented capsid targeting LR for viral assembly in Golgi complex, and budding through Golgi-derived vesicles. LRs serve as a platform for KSHV assembly. Mutation in ORF45 (K297R) results in immature virion particles that fail to target LR, but are degraded in lysosomes (Adapted from Wang et al., 2015).

Kaposi’s sarcoma-associated herpesvirus assembly and egress is a multiple-step process. Although the details of many steps in the process are largely unknown, the general model for herpesviral particle assembly is as follows: (1) Newly synthesized viral DNA is incorporated into preformed capsid in the nucleus; (2) intranuclear capsid leaves the nucleus by primary envelopment at the inner nuclear membrane and the primary envelope then fuses with the outer leaflet of the nuclear membrane, releasing capsids into the cytoplasm; (3) capsids acquire tegument in the cytoplasm; (4) final envelopment occurs by budding into Golgi-derived vesicles; (5) mature virions are released after fusion of the vesicle membrane with the plasma membrane of the cell (reviewed in Mettenleiter, 2002; Mettenleiter et al., 2009). This model appears to be true for KSHV virion assembly (**Figure 5**).

### Formation of Nucleocapsids and Nuclear Egress of KSHV Capsid

All herpesviruses including KSHV have thick-walled icosahedral nucleocapsids, ∼125 nM in diameter. The nucleocapsid of KSHV is composed of five capsid proteins, namely the major capsid protein (MCP) in hexameric and pentameric capsomers; triplex proteins 1 and 2 (TRI-1 and TRI-2, respectively) forming heterotrimers in a 1:2 ratio to connect the capsomers; the smallest capsid protein (SCP) stabilizing the capsid by crosslinking neighboring subunits of the MCP of hexons (Dai et al., 2015), and the portal protein (ORF43) forming a dodecameric structure for DNA genome packaging at one of the 12 icosahedral vertices (Trus et al., 2001). The assembly of herpesvirus capsid appears to be a spontaneous process, as the capsid of HSV-1 (herpes simplex virus 1, an alphaherpesvirus) can be assembled in a cell-free system (Newcomb et al., 1994, 1996). Assembly of herpesviral procapsid may initiate at aggregation of capsid proteins to produce protomers, which then assemble into higher-order procapsid structures (Aksyuk et al., 2015). It is likely to be true with KSHV capsids.

After the procapsid is formed, newly synthesized viral genomic DNA enters it through a DNA-packaging/ejection portal complex located at only one of the vertices of the capsid (Deng et al., 2007). The DNA-packaging/ejection portal complex is composed of a portal protein (PORT), which is encoded by ORF43 in the KSHV genome.

Like all herpesviruses, KSHV capsids are formed in the nucleus of infected cells, while final maturation occurs in the cytoplasm. To access the final assembly compartment, intranuclear capsids have to cross the nuclear envelope barrier. The current model for nuclear egress of herpesviral capsid is the envelopment-deenvelopment-reenvelopment model (Mettenleiter, 2002). In this model, mature nucleocapsids undergo a process of primary envelopment through the inner nuclear membrane and bud into the perinuclear space as enveloped viral particles. This is followed by deenvelopment at the outer nuclear membrane, releasing unenveloped capsids in the cytoplasm. Herpesviruses may usurp cellular endosomal sorting complex required for transport (ESCRT) machinery for nuclear egress (Lee et al., 2012), but the involvement of ESCRT in KSHV nuclear egress has not yet been proven.

### Tegument Proteins and Tegumentation

After nuclear egress, KSHV capsids acquire tegument proteins in the cytoplasm, which will be the tegument in the mature virion particles. The tegument used to be considered as an amorphous layer of proteins, but recent studies indicated ordered tegument structures in HSV-1 (Zhou et al., 1999), HCMV (Chen et al., 1999), MHV-68 (Dai et al., 2008), and KSHV (Dai et al., 2014). In *de novo* infection, tegument proteins play roles in viral entry, gene expression, and immune evasion. KSHV tegument protein ORF45 was demonstrated to antagonize innate immunity of host cells against the virus by blocking IRF-7 activation and nuclear translocation during *de novo* infection (Zhu et al., 2002, 2010; Sathish and Yuan, 2011). ORF52 subverts cytosolic DNA sensing by directly inhibiting cGAS enzymatic activity through a mechanism involving both cGAS binding and DNA binding (Wu et al., 2015b).

The tegumentation process is believed to be autocatalyzed through dynamic and intricate interactions amongst tegument protein themselves as well as with capsid. Composition of KSHV virions were analyzed using the state of the art proteomics approaches (Bechtel et al., 2005; Zhu et al., 2005). Interactions of the KSHV tegument proteins with different virion proteins have been systematically investigated, which led to the revelation of a virion-wide protein interaction network (Rozen et al., 2008). ORF64 encodes a 290-kDa tegument protein and the protein interaction map indicates a diverse interaction potential of ORF64 with many virion proteins. They include three viral capsid proteins, namely MCP, Triplex 1 and 2 (TRI-1 and TRI-2), suggesting an attachment of ORF64 to the icosahedral capsid structure (Rozen et al., 2008). A three-dimensional image reconstructed from a cryo-electron microscopic study of KSHV tegumented capsid particles revealed a density of three helices bundle that is distributed around the capsid vertices and interacts with penton and surrounding triplexes. The visualized filamentous material is likely to be ORF64 (Dai et al., 2014), consistent with the finding from the protein interaction study of ORF64 (Rozen et al., 2008).

Kaposi’s sarcoma-associated herpesvirus ORF64 also interacts with a number of tegument proteins including ORFs 11, 21, 33, 45, 63, 75 and itself, leading to a hypothesis that ORF64 is a scaffold protein, functioning as a major hub and recruiting other tegument proteins during KSHV tegumentation. In addition, ORF64 also interacts with some envelope glycoproteins (gB, gM, and gH) (Rozen et al., 2008). This finding justifies a rather peculiar behavior of ORF64 wherein it can be degraded when intact virion particles are treated with trypsin, suggesting the association of ORF64 with the viral envelope (Zhu et al., 2005). The interaction of ORF64 with viral glycoproteins is consistent with a hypothetical role of this protein in positioning the DNA-filled viral particles at the Golgi-derived vesicles through interaction with envelope glycoproteins, thus facilitating secondary envelopment processes.

Roles of other tegument proteins in tegumentation processes have been reported one after another in KSHV as well as related gamma-herpesviruses such as MHV-68. ORF33, ORF38, and ORF52 were all found to participate in tegumentation as deficiency of each of these proteins results in change of viral particle components. Loss of ORF33 or ORF38 in null-mutant recombinant viruses abolishes the recruitment of ORF45 into the KSHV and MHV-68 tegument (Guo et al., 2009; Wu et al., 2015a). ORF52-null recombinant KSHV and MHV-68 exhibited partially tegumented capsids with the absence of ORF45 (Bortz et al., 2007; Wang et al., 2012; Li et al., 2016). In summary, KSHV tegumentation is a self-assembly process following an intricate network of protein–protein interactions.

### Final Envelopment and Egress

Kaposi’s sarcoma-associated herpesvirus particles acquire their membranous envelope by budding into the lumen of cytoplasmic vesicles. This process is initiated by viral components, which recognize the budding site and recruit cellular cargo transport and sorting machinery to the site to complete the budding process. Herpesvirus budding and virion maturation, especially for gamma-herpesviruses, is poorly characterized. However, recent progress has demonstrated the roles of KSHV tegument proteins in orchestrating the final envelopment and budding process (Wang et al., 2015).

As mentioned above, ORF64 associates with the viral capsid, demonstrated by a 5Å cryoEM structure of KSHV tegumented capsid (Dai et al., 2014) as well as by the interactions between KSHV ORF64 and three capsid proteins (MCP, TRI-1, and TRI-2) previously observed (Rozen et al., 2008). Other evidence suggests that ORF64 also interacts with virion envelope membrane. Thus, it is speculated that ORF64 fulfills the roles of other viral matrix proteins by interacting with the capsid with one end and attaching the viral envelope with the other end, therefore suggesting a role of ORF64 in positioning DNA-filled capsid on the membrane of TGN where viral envelopment occurs.

Then, how does a KSHV particle bud through the membranous organelle and thereby acquire its membrane? Enveloped virus budding is topologically equivalent to cellular vesiculation process (endocytosis) and multivesicular body formation (**Figure 5**). It is believed that herpesviruses usurps cellular membrane sorting and transport machinery to execute the budding process like HIV-1 that was shown to utilize cellular ESCRT for its budding from the plasma membrane (Votteler and Sundquist, 2013). In spite of involvement of cellular machinery in viral budding, the process has to be initiated and orchestrated by viral component(s). Recently ORF45, as an outer tegument protein in KSHV particles, was found to be responsible for co-localization of viral particles with cytoplasmic membrane vesicles that carry *trans*-Golgi and endosome markers (TGN46 and EEA1) and facilitates the budding of viral particles into the membrane vesicles and the release of virion particles. The ability of ORF45 to target cytoplasmic vesicle at lipid raft (LR) domain is dependent on the mono-ubiquitylation of ORF45 at Lys297. Mutation at Lys297 (K297R) impairs ORF45 and viral particle co-localization with *trans*-Golgi network and endosomes (**Figure 5**). Furthermore, the recombinant KSHV carrying K297R mutant ORF45 (BAC-K297R) was severely defective in producing mature and infectious virion particles in comparison to wild-type KSHV (Wang et al., 2015). This finding revealed a function of ORF45 in targeting cytoplasmic membrane vesicles (likely *trans*-Golgi network or derived vesicles), facilitating the maturation and release of virion particles.

## KSHV Lytic Cycle and Oncogenesis

A traditional concept was that the lytic cycle of a tumor virus presumably does not contribute to oncogenesis simply because the lytic cycle in general leads to lysis and death of host cells. However, accumulative evidence suggests that the KSHV lytic cycle appears to be crucial for tumorigenesis. The most compelling evidence for this notion is that regression of KS lesions or decrease in the incidence of KS development were observed after KS patients or AIDS patients at risk for KS were treated with anti-herpesviral drugs, such as ganciclovir and foscarnet, that inhibit viral lytic but not latent replication (Martin et al., 1999). Furthermore, KSHV lytic replication spreads viruses to target cells and provides paracrine regulation, contributing to tumorigenesis (Cesarman et al., 2000). In addition, a new role of lytic replication has been described in sustaining the population of latently infected cells that otherwise would be quickly lost by segregation of latent viral episomes as spindle cells divide (Grundhoff and Ganem, 2004). Thus, KSHV lytic replication and constant infection to fresh cells are crucial for viral pathogenesis and tumorigenicity.

Among the potential oncogenes identified in the KSHV genome, many are classified in the category of viral lytic genes and have been shown to primarily express in the lytic life cycle. K1 encodes a type I membrane proteins and expresses in lytic early kinetics. Its expression can be detected in KS, PEL, and multicentric Castleman’s disease (MCD). Transgenic mice expressing the K1 gene exhibited constitutive activation of NF-κB and Oct-2, increased Lyn tyrosine kinase phosphorylation and elevated bFGF expression. Some of these mice developed spindle cell sarcomatoid tumor and plasmablastoid lymphoma (Prakash et al., 2002). ORF74-encoded vGPCR also expresses in the lytic phase with the early kinetics. vGPCR transgenic mice developed KS-like angiogenic lesions (Yang et al., 2000; Guo et al., 2003; Montaner et al., 2003). K2 encodes a vIL-6, which can be detected in MCD, PEL, and KS to different extents. vIL-6 signaling results in increased VEGF expression and vascularized tumors in mice (Aoki et al., 2001; Liu et al., 2001).

The paradoxical roles of KSHV lytic cycle and lytic proteins in tumorigenesis are still elusive but can be considered in several possibilities: (1) KSHV may not effectively immortalize infected cells or provide cell growth advantage *in vivo* (as observed *in vitro* in cell culture conditions). Spontaneous lytic replication of a small portion of infected cells in a lesion or population pool is critical for maintenance of infected cell populations by replacing dead cells or the cells whose viral genome is lost with freshly infected cells; (2) It is possible that some lytic proteins are expressed outside of the traditional lytic cycle and abortive replication life cycle allows specific lytic genes to be expressed in the absence of fully lytic replication; (3) Viral lytic replication may trigger activation of cellular oncogenic pathways or those for proliferation and inflammation, which contributes to KSHV oncogenicity in a greater extent than the latency-associated oncogenic potential. This notion is supported by the observation that EBV lytic genes are coexpressed with cellular cancer-associated pathways revealed by an EBV transcriptome study (Arvey et al., 2012).

## Future Perspectives

The two life cycles (latency and lytic replication) of herpesviruses and regulated switches between them represent extremely complex mechanisms that the viruses developed in co-evolution with hosts for millions of years. This allows herpesviruses to achieve both persistent infection for lifetime of the host and efficient dissemination from its long-term reservoir to the sites of disease or of shedding to spread to new hosts. Although we have learned a lot as summarized in this review regarding these two life cycles of KSHV and the mechanisms that control the switch between the two cycles, it might be just a tip of an iceberg. Many important questions remain elusive but progress is expected in the following aspects.

First, the regulatory mechanisms that control the switch between the two viral life cycles are complex and sophisticated. New categories of regulators and novel layers of control mechanisms are expected to emerge. Revelation of PAN RNA in epigenetic regulation of KSHV reactivation (Rossetto et al., 2013; Rossetto and Pari, 2014) is likely to be an overture that will be followed by a new chapter of regulation of viral latency and reactivation by non-coding RNAs, both viral and cellular. Furthermore, the microPeptide-mediated manipulation of protein function that adds a new dimension of biological regulation is on the horizon (Jaber and Yuan, 2013).

Second, increasing evidence suggests that contributions of KSHV lytic life cycle to oncogenesis are far greater than what we thought before. Is KSHV reactivation and lytic replication required for tumorigenesis by increasing viral titer to facilitate viral dissemination to the sites of tumor? At least 12 KSHV lytic proteins have been demonstrated to involve oncogenesis including transforming, pro-angiogenic, pro-growth, anti-apoptotic, or immuno-modulatory functions. Do these viral lytic proteins thus serve as direct effectors of the disease phenotypes and do lytically infected cells serve not only as reservoirs of infectious virus but also as reservoirs of pathogenic viral proteins? Research on these questions will provide novel insights into pathogenesis of tumor viruses.

Third, KSHV lytic replication can be considered to design therapeutic strategies for treatment and prevention of KS due to its etiological role in KS of all types (classic KS, endemic KS, AIDS-associated KS, and IRIS-KS in the HAART era). There is currently no definitive cure for KS. Classic cancer therapies are generally used to treat KS patients, which include surgical excision and radiation therapy for patients with a few lesions in a limited area and chemotherapy for patients with extensive or recurrent KS (Antman and Chang, 2000). The chemotherapeutics, which have been approved by the FDA and are often used, include liposomal anthracycline products (liposomal doxorubicin or liposomal daunorubicin), paclitaxel, and interferon-α (Potthoff and Brockmeyer, 2007). However, these therapeutic agents do not target the etiological virus and the tumor response to any chemotherapeutic regimen is only transient. In AIDS-associated KS, HAART regimens are associated with regression in the size and number of existing KS lesions (Lebbe et al., 1998). Despite its dramatic decrease in frequency since the advent of HAART, KS remains the most common AIDS-associated cancer (Boshoff and Weiss, 2002; Shiels et al., 2011). In addition, as experience with HAART has grown, a new HAART-associated syndrome, namely immune reconstitution inflammatory syndrome or IRIS, emerged. In a subset of HIV-seropositive individuals, initiation of HAART in the setting of advanced HIV infection results in a paradoxical clinical worsening of existing infection or the appearance of a new condition including KS in a process (Shelburne and Hamill, 2003). Recently new cases of pulmonary KS as a result of non-compliance HAART therapy have been reported (Epelbaum et al., 2016). As IRIS-KS is the result of responses by a recovered immune system to KS-causing pathogen, i.e., KSHV, treatment of KSHV-seropositive, HIV-positive patients with a combination of antiretroviral (HAART) and anti-KSHV chemotherapeutics is expected to yield positive results. However, currently there are no effective drugs targeting KSHV available. The recent progress in understanding of KSHV reactivation provides opportunities to develop targeted drugs against KSHV lytic replication. For example, RTA is unique transcription factor. Its DNA binding region does not contain any well-characterized DNA binding motifs and the overall protein shows no homology with any known cellular transcriptional activators. Therefore, RTA is an attractive target for antiviral therapy. In addition, virion assembly and egress processes are expected to be effective targets for broad-spectrum antiviral drugs.

## Author Contributions

KA and YY contribute to the writing, figure-making, and proofreading for this review article.

## Conflict of Interest Statement

The authors declare that the research was conducted in the absence of any commercial or financial relationships that could be construed as a potential conflict of interest.
